# Quantifying Age-Related Changes in Brain and Behavior: A Longitudinal versus Cross-Sectional Approach

**DOI:** 10.1523/ENEURO.0273-21.2021

**Published:** 2021-08-04

**Authors:** Georgette Argiris, Yaakov Stern, Christian Habeck

**Affiliations:** 1 Cognitive Neuroscience Division, Department of Neurology, Columbia University Medical Center, New York, New York 10032; 2 Taub Institute for Research on Alzheimer's Disease and the Aging Brain, Columbia University, New York, New York 10032

**Keywords:** cognitive aging, longitudinal, reference ability neural networks, within-subjects fMRI

## Abstract

Cross-sectional versus longitudinal comparisons of age-related change have often revealed differing results. In the current study, we used within-subject task-based fMRI to investigate changes in voxel-based activations and behavioral performance across the life span in the Reference Ability Neural Network cohort, at both baseline and 5 year follow-up. We analyzed fMRI data from between 127 and 159 participants (20–80 years) on a battery of tests relating to each of four cognitive reference abilities. We applied a Gaussian age kernel to capture continuous change across the life span using a 5 year sliding window centered on each age in our participant sample, with a subsequent division into young, middle, and old age brackets. This method was applied separately to both cross-sectional approximations of change and real longitudinal changes adopting a comparative approach. We then focused on longitudinal measurements of neural change to identify regions expressing peak changes and fluctuations of sign change across our sample. Our results revealed several regions expressing divergence between cross-sectional and longitudinal measurements in each domain and age bracket; behavioral comparisons between measurements showed differences in change curves for all four domains, with processing speed displaying the steepest declines. In the longitudinal change measurement, we found lack of support for age-related frontal increases across analysis types, instead finding more posterior regions displaying peak increases in activation, particularly in the old age bracket. Our findings encourage greater focus on longitudinal measurements of age-related changes, which display appreciable differences from cross-sectional approximations.

## Significance Statement

Knowledge of the aging process is mostly informed by cross-sectional studies. The fewer studies that have looked at longitudinal aging trajectories display variable consensus with cross-sectional findings. The current study provides a direct comparison between cross-sectional and longitudinal measurements of change in both neural activation and behavioral performance across several cognitive domains, providing insight into similarities versus discrepancies. Furthermore, it adopts a method of analysis used in the MRI 4D atlas literature to quantify continuous change across the life span through construction of neural activation “templates” that are generated from age-weighted averaging across the entire sample. Longitudinal measurements of change could then be probed further for characteristics such as peaks and change fluctuations, enabling a better understanding of true age-related changes.

## Introduction

Cognitive functions and their underlying neural substrates change across the life span (for review, see Grady, 2012). Cross-sectional measurements of these changes often reveal a decline in behavioral performance across several domains including reductions in general processing speed (Salthouse, 1998), episodic memory (Tulving, 2002), fluid intelligence (Kievit et al., 2014), visuospatial and verbal working memory (Cansino et al., 2013), long-term memory (Park et al., 2002), selective attention (Madden, 2007), and task switching (Wasylyshyn et al., 2011), among others. Conversely, some aspects of cognition, such as semantic priming (Laver, 2009), are shown to remain intact or even increase with age, such as vocabulary (Salthouse, 2014a; Hartshorne and Germine, 2015). However, cross-sectional versus longitudinal comparisons have revealed different patterns of age-related changes; whereas the former often reports monotonic declines beginning as early as the 20s (Salthouse, 2014b), the latter shows a preservation of function until later in life, with older adults displaying an accelerated slope of decline in domains such as fluid reasoning (De Vis et al., 2018), memory (Salthouse, 2019), and global cognition (Singh-Manoux et al., 2011). Furthermore, a recent longitudinal meta-analysis by Tucker-Drob et al. (2009) found support for age-related increases in the shared variance of change across cognitive domains because of purported increased reliance on a common underlying factor (e.g., the g factor).

At the neural level, changes in brain activation from young to old adulthood have mainly been studied cross-sectionally and have yielded variable results. Some studies have observed reduced brain activity in older compared with younger adults, which has often been interpreted as a deficiency of processing, particularly when it is linked to reduced behavioral performance (Grady et al., 1995; Rypma and D'Esposito, 2000). Conversely, other studies have observed age-related increases in brain activity, which has often been linked to compensatory processing mechanisms (for review, see Eyler et al., 2011). One prominent theory endorses a posterior–anterior shift with aging (Davis et al., 2008), where greater age-related activation is reported in prefrontal cortical regions and reduced activation on memory tasks (Cabeza et al., 1997, 2004; Reuter-Lorenz et al., 2000). A compensatory interpretation has accompanied diverse behavioral outcomes, such as increased activation among older adults who perform comparably to their younger counterparts (Cabeza et al., 2002), when positive correlations between performance and activation selectively occur in older adults (Grady et al., 2005), or even in the presence of impaired performance among older adults (Zarahn et al., 2007). Together, these studies have suggested that older adults typically use neural resources in prefrontal cortex (PFC) regions to buffer against the adverse impact of aging with the goal of aiding/maintaining performance.

Compared with the wealth of cross-sectional studies comparing age groups, fewer studies have focused on the intraindividual longitudinal changes that occur with age, largely because of methodological limitations such as attrition and measurement “impurities” introduced by practice effects. A good portion of the longitudinal studies that do exist has been concentrated on the episodic memory domain. Results have varied, from memory performance remaining stable over the testing period despite functional alterations in cerebral blood flow (Beason-Held et al., 2008a,b), to successful agers displaying higher fMRI BOLD activation in the left hippocampus and bilateral PFC (Pudas et al., 2013), to memory decline being linked to increases in PFC activation and reduction in right hippocampal volume (Pudas et al., 2018); fluctuations in hippocampal activation across testing sessions have also been linked to an increased slope of cognitive decline (O’Brien et al., 2010). Whereas longitudinal studies of behavioral changes have broached different cognitive domains, such as processing speed, and crystallized and fluid ability (for review, see Ghisletta and Lindenberger, 2004), and even their link to protective factors in buffering decline (Manly et al., 2003; Tucker-Drob et al., 2009; Then et al., 2015), fewer studies have comprehensively addressed the neural changes that accompany healthy aging across different domains.

In the present study, we use longitudinal data from the Reference Ability Neural Network (RANN) study to derive both cross-sectional approximations of change across the life span as well as actual longitudinal measurements of change over a 5 year span. To characterize age-related change, we applied a Gaussian kernel across the ages in our sample to generate both (1) weighted neural activation maps of change as well as (2) weighted behavioral scores across a sliding 5 year window. This allowed us to generate “templates” of change, which is a concept borrowed from the MRI 4D atlas literature, where attention has been given to chronicling dynamic life span changes (Ericsson et al., 2008; Serag et al., 2012). This allowed us to also midlife changes, which has only recently garnered attention in the aging literature (Pudas et al., 2014; Hughes et al., 2018). We refrained from adopting a statistical approach such as mixed-effects modeling because our intention here was to avoid constraining our analyses to model-based assumptions and instead explore trends in the data in a more phenomenological vein. Given the novelty of our approach and application across multiple domains in a longitudinal dataset, we refrained from making strong a priori claims. Instead, we merely hypothesize that several regions will show insightful discrepancies between real longitudinal measurements of change and cross-sectional approximations of such change, and that areas of maximal change across time and space will differ by domain.

## Materials and Methods

### Participants

A sample size of between 127 and 159 participants, depending on the domain, was included in the analysis (Table 1, list of participant demographics). As we wanted to maximize participant inclusion, we did not restrict our sample to only those participants who completed all 12 tasks of our design; we treated each domain separately, which accounted for the varying sample size. A participant was only required to have data for at least one task in a given domain. All participants were native English speaking, right handed (Oldfield Edinburgh Handedness Inventory; Oldfield, 1971) adults who were tested at two time points—baseline and 5 year follow-up—with an age range of 20–80 years at baseline. Participants were recruited for the study via random market advertising. All participants were screened for severe medical or psychiatric conditions, head injury, hearing or vision impairments, and other impediments that could interfere with MRI acquisition. Older participants were screened for dementia and mild cognitive impairment using the Dementia Rating Scale (Mattis, 1988) at both time points. All participants had <30% of their data “scrubbed,” explained in the fMRI data preprocessing section.

**Table 1:** Participant demographics divided by age bracket

			Sex		Age		NART		Education	
Age bracket	Domain	*N*	Male	Female	Mean	SD	Mean	SD	Mean	SD
20–40 years	MEM	40	16	24	30.83	5.75	112.94	7.81	16.33	2.26
	FLUID	47	18	29	31.19	5.55	112.07	7.73	16.17	2.51
	SPEED	50	18	32	30.52	5.49	112.73	7.68	16.04	2.47
	VOCAB	49	18	31	30.7	5.4	112.71	7.76	16.04	2.5
41–60 years	MEM	40	18	22	50.58	5.69	119.42	7.36	16.15	2.34
	FLUID	42	19	23	50.48	5.57	117.94	7.83	15.95	2.25
	SPEED	49	24	25	49.9	5.71	118.93	7.78	16.02	2.33
	VOCAB	46	22	24	49.57	5.66	118.71	7.93	15.98	2.31
61–75 years	MEM	47	25	22	68.11	5.21	119.4	7.43	16.51	2.52
	FLUID	59	31	28	68.71	4.89	119.57	7.47	16.56	2.62
	SPEED	60	32	28	67.97	5.06	119.69	7.39	16.56	2.6
	VOCAB	57	31	26	68.23	5.08	119.7	7.06	16.47	2.67

Age, NART (National Adult Reading Test) score, and education represent values at baseline. Counts (*N*) are given for the total number of participants in each domain, along with a division by sex.

### Procedure

The experiment was designed to acquire fMRI data from participants as they performed 12 computerized cognitive tasks in the scanner, each relating to one of four reference abilities (RAs; Stern et al., 2014), at two time points (baseline and 5 year follow-up). At each testing time point, participants completed the battery of tasks over two sessions, each lasting for ∼2 h and containing 6 of the 12 tasks belonging to two of the four RAs. Tasks within each reference domain were presented in a fixed order; the order of the two sessions was counterbalanced across participants. The order of administration at follow-up was completely randomized and did not depend on the order of administration at baseline. Tasks presented at follow-up were identical to those presented at baseline. As previously mentioned, we treated each domain separately, and thus participants were only required to have performed at least one of the tasks in a given domain to be included in the analysis. This was done to maximize participant inclusion considering the difficulty of procuring complete sets of longitudinal data. Therefore, the number of participants in each domain varies. To ensure that there was no difference in the number of tasks completed as a function of age, we pooled together participants across all domains (184 participants in total) and compared the total number of tasks completed between age brackets, for both baseline and follow-up. One-way ANOVA revealed no significant difference between age brackets, either at baseline (*F*_(1,182)_ = 1.03, *p* = 0.31) or follow-up (*F*_(1,182)_ = 0.826, *p* = 0.36). The mean number of tasks completed (baseline/follow-up) were similar across young (11.69/10.9), middle (11.62/11.08), and old (11.84/10.6) age brackets.

Before each scanning session, participants were familiarized with the six tasks relevant to the current session during an out-of-scanner training session, which was performed on a laptop computer. The mode of response for all but one task was keyboard button press; the picture-naming task used an oral response. Training sessions were self-paced, such that breaks could be taken when needed, and participants were given the option of repeating the training session if desired. The assessment of task comprehension was made based on the participant’s subjective comfort with the task and the informed judgment of a trained research assistant. For the scanning portion, breaks were also permitted on request and could be taken between the completion of the cognitive tasks and the beginning of the structural scans; however, breaks were rarely requested. In a separate session, participants also completed a neuropsychological battery; results from this battery will not be addressed in the current article.

#### Stimulus presentation

Stimuli were back-projected onto an LCD monitor positioned at the end of the scanner bore. Participants viewed the screen via a tilted mirror system, which was mounted on the head coil. When needed, vision was corrected to normal using MR-compatible glasses (SafeVision). Responses were made on a LUMItouch response system (Photon Control). E-Prime version 2.08, operating on a PC platform, was used for stimulus delivery and data collection. Task onset was electronically synchronized with the MRI acquisition device.

#### Reference ability in-scanner tasks

Twelve cognitive tasks, each belonging to one of four reference domains, were presented in the scanner. A brief description of each task, divided by domain, is provided below (for a more thorough description, see Stern et al., 2014). For all tasks, with the exception of picture naming, responses were made via button press; picture naming, instead, required a vocal response. For episodic memory (MEM), fluid reasoning (FLUID), and vocabulary (VOCAB) domains, accuracy, measured as the proportion of correct trials to total trials included, was analyzed for each task. For the processing speed (SPEED) domain, response time data were analyzed for each task. For the remainder of the document, an abbreviated version for each reference ability will sometimes be used: MEM, FLUID, SPEED, and VOCAB. We also will interchangeably use the terms “domain” and “reference ability” to refer to our RAs.

##### MEM

For all three episodic memory tasks, both study and test phases were scanned together and cannot be separated in the analysis. The percentage of correct trials served as the behavioral variable of analysis. The tasks were as follows. (1) With logical memory, participants were presented with a story scenario on the computer screen. They were required to read the story and answer detailed multiple-choice questions regarding the content, choosing one of four possible answers. (2) In word order recognition, in the study phase, participants were presented with a list of 12 words, 1 word at a time, on the computer screen and asked to remember the order of word presentation. In the test phase, participants were presented with a probe word at the top of the screen and four choice words below, and were asked to indicate which of the four choice words was presented subsequent to the probe word. (3) For paired associates, in the study phase, participants were presented with a list of 12 word pairs, 1 pair at a time, on the computer screen and were asked to remember the word pairings. In the test phase, participants were presented with a probe word and four choice words below and asked to select which word was previously paired with the probe word.

##### FLUID

The percentage of correct trials served as the behavioral variable of analysis. The tasks were as follows. (1) In matrix reasoning (adapted from Raven, 1962), participants were presented with a matrix divided into nine cells (3 × 3) that reflected an unspecified rule, with the bottom right cell remaining empty. Participants had to decide which of eight figure choices, presented below the matrix, best completes the sequence pattern. (2) In letter sets (Ekstrom et al., 1976), participants were presented with five sets of letters, with four of them expressing a common rule (e.g., contains no vowels). Participants were asked to infer the rule and identify the letter set that deviates from it. (3) In paper folding (Ekstrom et al., 1976), participants were presented with a paper folded in a specific sequence with a set of holes punched through it. They had to decide which of six options reflected the configuration of the holes on the paper when unfolded.

##### SPEED

Reaction time served as the behavioral variable of analysis. The tasks were as follows. (1) In digit symbol (adapted from Salthouse, 1998), participants were presented with a code key at the top of the screen consisting of nine number (values ranging from one to nine)–symbol pairs. Below the code key, a single number–symbol pair was presented, and participants were asked to indicate whether the pair was present in the code key. (2) In letter comparison (Salthouse and Babcock, 1991), participants were presented with two strings of letters alongside one another, each containing three to five letters. They were asked to indicate whether the strings were the same or different. (3) In pattern comparison (Salthouse and Babcock, 1991), participants were presented with two figures alongside one another, each consisting of connected lines that formed different configurations. They were asked to indicate whether the figures were the same or different.

##### VOCAB

The percentage of correct trials served as the behavioral variable of analysis. The tasks were as follows. (1) With antonyms (Salthouse and Kersten, 1993), participants were presented with a probe word in capital letters at the top of the screen. Below the probe word, four choices of words were listed. They were asked to indicate which word possessed a meaning that was most dissimilar to that of the probe. (2) With picture naming, participants were presented with single images and asked to identify the picture by vocal response. Images were selected from the WJ-R Psycho-Educational battery (Woodcock et al., 1989; Salthouse, 1998). (3) With synonyms (Salthouse and Kersten, 1993), participants were presented with a probe word in capital letters at the top of the screen. Below the probe word, four choices of words were listed. They were asked to indicate which word possessed a meaning that was most similar to that of the probe.

#### fMRI data acquisition

Image acquisition was performed using a 3 T Philips Achieva Magnet scanner. Participants performed 12 fMRI tasks over the course of two 2 h MR imaging sessions; the same procedure was followed at both baseline and again at the 5 year follow-up. At the onset of each session, a scout T1-weighted image was acquired to determine the participant’s position. A T1-weighted MPRAGE scan was performed to capture participants’ brain structures, with the following parameters: TE, 3 ms; TR, 6.5 ms; flip angle, 8°; in-plane resolution, 256 × 256 voxels; field of view, 25.4 × 25.4 cm; and 165–180 slices in the axial direction; slice thickness, 1 mm; slice gap, 0 mm. All scans used a 240 mm field of view. For the EPI acquisition, the following parameters were used: TE, 20 ms; TR, 2000 ms; flip angle, 72°; in-plane resolution, 112 × 112 voxels; slice thickness, 3 mm; slice gap, 0 mm. FLAIR (fluid-attenuated inversion recovery), DTI (diffusion tensor imaging), ASL (arterial spin labeling), and a resting BOLD (7 min) scan were additionally acquired; however, these data are not considered in the current article. A neuroradiologist examined each participant’s scan for abnormality, and any significant findings were reported to the participant’s primary care physician.

#### fMRI data preprocessing

FMRIB Software Library (FSL) version 5.0 and custom-written Python code was used to preprocess the imaging data. The preprocessing pipeline for each participant’s task-related scan was performed using FSL (Smith et al., 2004) with the following steps: (1) generation of within-participant histograms for noise detection (FEAT); (2) spatial realignment to the middle volume (MCFLIRT); (3) slice timing correction; (4) creation of brain mask from the first volume; (5) high-pass filtering (T = 128 s); (6) prewhitening for attenuation of autocorrelation; (7) general linear model (GLM) estimation with motion-related nuisance regressors and convolved double-gamma hemodynamic response function (HRF); and (8) nonlinear registration of functional to structural brain images with normalization into MNI space (FNIRT).

#### Time-series modeling

For each participant, general linear models were created, consisting of block-based time series for fluid reasoning, speed and vocabulary tasks, and event-related models for the memory tasks. For the memory tasks, while the encoding, retention, and retrieval phases were imaged, only the retrieval phase was analyzed. A single regressor was used to compare task performance to an intrinsic baseline, which was defined in one of two ways depending on the analysis. For block design task models, a boxcar function denoting the onset and offset of each task block was used. The regressor was obtained by convolving this boxcar function with the canonical HRF. The intrinsic baseline was defined as the interval between task blocks during which no stimuli were presented on the screen. For event-related task models, the intrinsic baseline was modeled as the combination of all nontask periods. Each stimulus presentation was modeled from the onset of the stimulus to the response, using correct trials only, with the regressor obtained by convolving the stimulus presentation with the canonical HRF. For each participant’s 12 tasks, a standard GLM was run on each scan, using the appropriate regressor, to generate a parameter estimate (β) map. A gray matter mask was applied to the data to include only those voxels with a mean gray matter probability of ≥50% across all participants. This reduced the number of active voxels to 24,055. Analyses were performed on this masked subset.

### Analytical approach

Data were analyzed using custom-written MATLAB codes (MathWorks). For between-task comparison in behavioral performance, all scores were standardized via *z*-transformation, with the mean and SD calculated at the first visit across all participants for each task separately. For speed tasks, *z*-score values were sign inverted to correspond with accuracy scores from other task domains, such that higher scores always reflect better performance. For adequate comparisons between testing time points, *z*-score transformations of both baseline and follow-up data were made based on the mean and SDs calculated at baseline. For analyses of all behavioral and voxel-wise fMRI data at both baseline and follow-up, behavioral performance and activation maps, respectively, for the three tasks pertaining to a given domain were averaged. That is, for each participant, a single activation map per domain was first created by averaging across the tasks pertaining to each RANN domain.

### Age kernel

We were interested in ascertaining how domain-related activation changes across the life span, comparing cross-sectional approximations of change at baseline to real changes derived from the longitudinal data. We first explain how baseline data were analyzed, followed by longitudinal calculations of change.

We used an age kernel (explained in greater detail in the section to follow) to enable a finer-grained consideration of change as a function of the age of the participants: the age kernel. The kernel creates a weighted average, across all participants, of a measured phenomenon (i.e., neural activation or behavioral performance), enabling some age specificity by assigning greater weight to participants whose age falls closer to a particular target age. This is a compromise between averaging across all participants (no age specificity, but less statistical noise) and considering single participants only (great age specificity, but more statistical noise). As a first measurement of change in task-related activation, we applied our kernel across all voxels to generate change curves. We then followed this up with an application of the kernel to subsets of voxels selected from ROIs centered on each voxel in our mask.

#### Neural age-weighted maps of baseline data

##### Generation of age-weighted activation (β) maps

**Figure 1.**
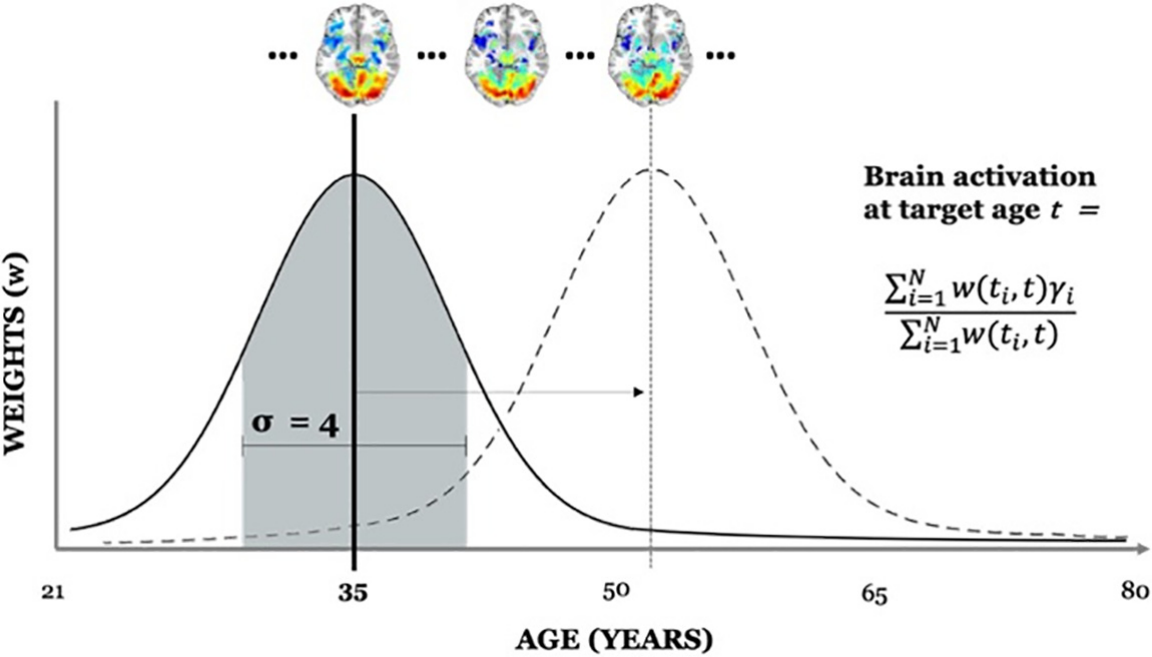
Schematic of the generation of weights defined by the Gaussian kernel (σ = 4) centered at a target age of 35 years old. Dashed Gaussian demonstrates the kernel sliding across and centered on each year of age present in the dataset. In the equation of brain activation at target age (*t*)*, w*(*t_i_, t*) is the weight (*w*) assigned to a participant’s age (*t_i_*) given the target age (*t*) and γ*_i_* is the participant’s domain activation (β) map. In the weighing of each map γ*_i_*, the voxel index is preserved. The example brains above demonstrate the resulting output, which is weighted activation maps at each year of life in the sample, for each of the four domains.

To investigate cross-sectional approximations of change across age in the baseline data, we used a Gaussian smoothing function to create activation maps at 1 year age increments by integrating across all participant’s age-weighted activation maps. The aim was to use each participant’s domain activation map, by weighting its signal, to generate a mean domain activation map for each target age. The weight, or the degree to which a participant’s signal contributed to the mean signal, depended on the participant’s age with respect to the target age. For a given domain and target age (*t*), the procedure was as follows. (1) We applied a Gaussian kernel to age, centered on a target age (*t*), to obtain a weight (*w*) for each participant’s age (*t_i_*). Weights were derived according to the Gaussian function, defined as follows:

$$w(t_{i},t)=\frac{1}{\sigma \sqrt{2\pi }}e^{\frac{-{\left(t_{i}-t\right)}^{2}}{2\sigma^{2}}}$$

where the width, or SD (σ), of the kernel is a somewhat subjective parameter determined by the size and distribution of the dataset; lower values of σ weigh the tails of the age distribution less, leading to a sharp localization around the target age, whereas higher values create a more dispersed “blunt” distributional spread. As we had a relatively large sample size, we followed the choice, σ = 4, of Ericsson et al. (2008) who, in their generation of a 4D structural atlas, found that good results could be obtained using 3 < σ < 5. Good results in their analysis were defined as not too heavily weighing individual samples yet not smoothing out over age-dependent variation, either of which could occur with too small or too large values of σ, respectively. To assess the reliability of our choice in σ given the range of this window, we also performed the kernel regression using σ values of 3 and 5. Similar results were obtained across these σ values. As a reminder, the kernel was centered on each age in our dataset, ranging from 20 to 80 years, with each age serving as a target age, and weights assigned to all participant’s ages accordingly. (2) After obtaining age weights for a target age (*t*), we multiplied each participant’s domain activation map by their age-defined weight to create a weighted map per participant. (3) We then summed these weighted maps across participants and divided by the sum of the weights to create a single mean activation map for the target age. An example of the kernel centered at target age (*t* = 35 years old) can be found in Figure 1. (4) The result was a weighted activation map (24,055 voxels) per year of life (61 time points: 20–80 years) for each of the four domains.

##### Activation map change curves estimated from cross-sectional data at baseline

We were interested in quantifying the change in age-weighted activation maps across time by deriving a single change in activation (Δ*A* value per 5 year sliding window; e.g., 20–25, 21–26). To do so, we subtracted the activation map at time *t* from the map at time *t *+* *5. Given the age span of 20–80 years at baseline, change maps could only be calculated up until 75–80 years, yielding 56 change maps per domain. We then took the mean across all voxels. This resulted in a 56 × 1 vector of Δ*A* values per domain, with positive values reflecting 5 year age-related increases in activation and negative values reflecting 5 year age-related decreases in activation.

##### Age-weighted activation ROIs and change curves estimated from cross-sectional data at baseline

We also wanted to obtain a more refined and precise measure of age-related change across the brain. To do so, rather than generating age-weighted maps per year of life by summing across all participant age-weighted activation maps (24,055 weighted voxels), we generated 24,055 ROI spheres, centered on each voxel, and age weighted the subset of voxels comprised by each ROI. ROIs were generated by centering a 12-mm-radius sphere on each voxel in our gray matter mask and selecting those voxels that fell within this sphere. Because of the irregularity of the gray matter mask, voxel count by ROI varied (median, 193 voxels; range, 16–428 voxels). Per ROI, we first obtained the index of voxels corresponding to a given ROI and selected only those voxels from participants’ domain activation maps. Next, for each target age, we multiplied each participant’s voxel activation values by their age-defined weight (corresponding to step 2 above) and then summed across all participants and divided by the sum of the weights (corresponding to step three above); this yielded a weighted ROI (between 16 and 428 voxels) per year of life (61 time points: 20–80 years) for each of the domains (four). To create the change curves, for each ROI of each domain, we subtracted the ROI voxel activation values at *t* from the map at *t *+* *5 and averaged across all voxels comprising that ROI. This rendered a 56 × 1 vector of Δ*A* values per ROI (24,055), per domain (four).

#### Neural age-weighted change maps of longitudinal data

##### Generation of age-weighted activation change maps

To generate longitudinal change maps and their subsequent change curves, we inverted the process described above: instead of averaging across participants with the age kernel and then subtracting between different target ages, we now subtracted the activation maps at *t* from *t + *5 for each participant first, and then applied the kernel to create age-weighted change maps at each 5 year sliding window.

For a given domain and target age (*t*), the procedure was as follows. (1) We first calculated the difference between the activation map at *t* and the map at *t* + 5 within each participant. (2) Next, for each target age “interval” (e.g., 20–25 years), we created age-weighted change maps by multiplying each participant’s domain activation change map by their age-defined weight to create a weighted change map per participant. The weight assigned corresponded to their age at baseline. (3) As before, we then summed these weighted change maps across participants and divided by the sum of the weights to create a single mean activation difference map for the target age interval. (4) The result was a weighted activation change map (24,055 voxels) per sliding 5 year window of life (61 time points: 20–80 years) for each of the domains (four), centered on age at baseline. However, to render the longitudinal results comparable to the baseline results, we only considered the change maps between 20 and 75 years (the latter corresponding to the age interval of 75–80 years), resulting in 56 time points.

##### Activation map change curves of longitudinal measurements

To generate change curves, we again took the mean across all voxels. As before, this resulted in a 56 × 1 vector of Δ*A* values per domain.

##### Age-weighted activation ROIs and change curves of longitudinal measurements

We followed a procedure similar to the one described for the baseline approximation only inverting the weighting and subtraction steps. For the longitudinal differences, for each target age, per ROI, per domain, we first subtracted a participant’s ROI at baseline time (*t*) from that at follow-up time (*t *+* *5). We then age weighted these difference values and averaged them across all voxels within that ROI, again generating a 56 × 1 vector of Δ*A* values per ROI (24,055), per domain (four).

#### Age-weighted behavioral performance scores

##### Baseline approximation change curves

We applied the same Gaussian age kernel procedure as described above to behavioral performance to additionally observe how it changes across the life span. The same weights were generated for each target age, only this time, instead of multiplying the age-defined weight by the participant’s activation map, we multiplied it by the participant’s performance. As before, for each target age nested within each domain, the age-weighted performance scores were summed across all participants and divided by the sum of the weights. This yielded a single behavioral value for each target age (61 age points: 20–80 years) for each of the domains (four). As before, we were interested in quantifying the change in age-weighted behavioral performance across age by deriving a single change in performance (Δ*P*) value between each year. For each domain, we subtracted the weighted performance score at *t* from the score at *t *+* *5, yielding a 56 × 1 vector of Δ*P* values per domain.

##### Longitudinal change curves

Longitudinal change scores were calculated by first subtracting each participant’s performance at *t* from their performance at follow-up time (*t *+* *5). For each domain, we next calculated the performance change score per target age by multiplying each of the participant’s change values by the weight assigned to their age at baseline with respect to the given target age. We then summed across all participants per target age, which yielded a 61 × 1 vector of Δ*P* values per domain. To render the longitudinal results comparable to the baseline results, we only considered the change values between 20 and 75 years (the latter corresponding to the age interval of 75–80 years), resulting in 56 time points.

#### Comparisons between baseline approximations and longitudinal neural change: change curves per ROI divided by age bracket

We next wanted to compare baseline to longitudinal measurements of change between each ROI to see where the differences curves were most similar and most different to one another; that is, where baseline approximations adequately capture true changes and where there is high discrepancy between the two. To do this, we divided curves into age brackets comprising young age, middle age, and old age. Such a division was motivated by the idea that middle adulthood is often an overlooked time span in the aging literature, with comparisons typically focusing on extreme ends of the age distribution, and we wanted to take advantage of the expanse of our dataset. We defined young age as 20–40 years, which reflects the changes in activation over the period of 20/25–40/45 years; middle age as 41–60 years, covering the change interval of 41/46–60/65 years; and old age as 61–75 years, covering the change interval of 61/66–75/80 years. Our rationale for such age boundaries was determined by a few factors. We specifically defined older age as the period comprising 60–80 years based on prior literature (Reuter-Lorenz and Park, 2010). As for the young and middle age brackets, as previously mentioned, given the limited number of studies investigating midlife changes, there is not a stable precedence to follow that delineates the transition from young to middle adulthood. Therefore, we relied on the age distribution of our sample population and the few examples from the literature explicitly testing a middle age sample. Placing a boundary at 40 years of age allowed us to create rather evenly distributed tertile intervals, with the addition of having some founding in the literature (Ankudowich et al., 2016). Next, for each ROI (24,055) and domain (four), we compared segments of the two change curves comprising each of the three age brackets separately by computing the mean absolute error (MAE), which measures the average error between paired observations expressing the same phenomenon, regardless of the direction. It is calculated by simply subtracting one curve from the other and taking the mean of the absolute value of the differences. This rendered a map (24,055 ROI values) of MAE values per age bracket (three), per domain (four). To assess areas of high similarity or difference, we ultimately considered only those values falling beyond the 2.5 or 97.5 percentiles of the distribution, respectively.

#### Longitudinal change curves per ROI

As the longitudinal change curves reflect the true changes that occur over a 5 year age span, we chose to focus the rest of the analyses on ROI regions of maximum signed change in the longitudinal measurement only.

##### Integrated change by age bracket

We were interested in the areas exhibiting maximum change, in terms of both increases and decreases in activation, across the life span. We therefore calculated the integral of change values on segments of the change curves comprising each of the three age brackets, separately, per ROI (24,055) and domain (four). For each age bracket segment of the change curve, we first divided them into negative and positive change values to distinguish between cumulative increases versus decreases in activation. We then calculated the integral, or the area under the curve, for change values of each sign. The integral method that we used was trapezoidal, which approximates the area of the region between two units, or as in our case between two age intervals (e.g., 21/26–22/27), for each of the partitioned age intervals by essentially treating the difference between each age interval as a trapezoid and calculating its area. The integral over the entire age-bracketed segment is achieved by summing across the areas of each age interval. In this way, we obtained two total change values, reflecting positive or negative change, for each age bracket. As this procedure was performed per ROI and domain, we thus obtained maps (24,055 ROI values) for each domain (4), each age bracket (three), and each sign of change (two). As before, we were mainly interested in establishing which areas displayed extreme activation increases or decreases in each age bracket. Thus, we considered only those values that fell beyond the 97.5 percentile upper bound of increases in activation (positive) and beyond the 2.5 percentile lower bound of decreases in activation (negative).

##### Peak change across the life span

We also wished to establish the age bracket in which a peak change across the life span occurred for each of the ROI change curves in each domain. For each ROI in each domain, we located when a peak positive maximum and a peak negative change occurred in the change curve. We then color coded them by age bracket and generated peak maps (24,055 ROI peak values) per domain (four) that reflected the age bracket assignment.

##### Age dependence variability of longitudinal change

Last, we measured the variability in the direction of change in the longitudinal measurements across all domains, separately for each age bracket. That is, we wished to see which voxels fluctuated in the sign of change across all four domains. For each age bracket, we indexed when a voxel displayed at least one change of sign (i.e., zero crossing) in each domain and mapped those voxels displaying overlap across all domains. For example, imagine that a voxel shows at least one zero-crossing (i.e., sign fluctuation) in the young age bracket in each of the four domains. This voxel would be indexed and shown as “consistently variable change” according to our definition.

## Results

### Neural change curves averaged across voxels

**Figure 2.**
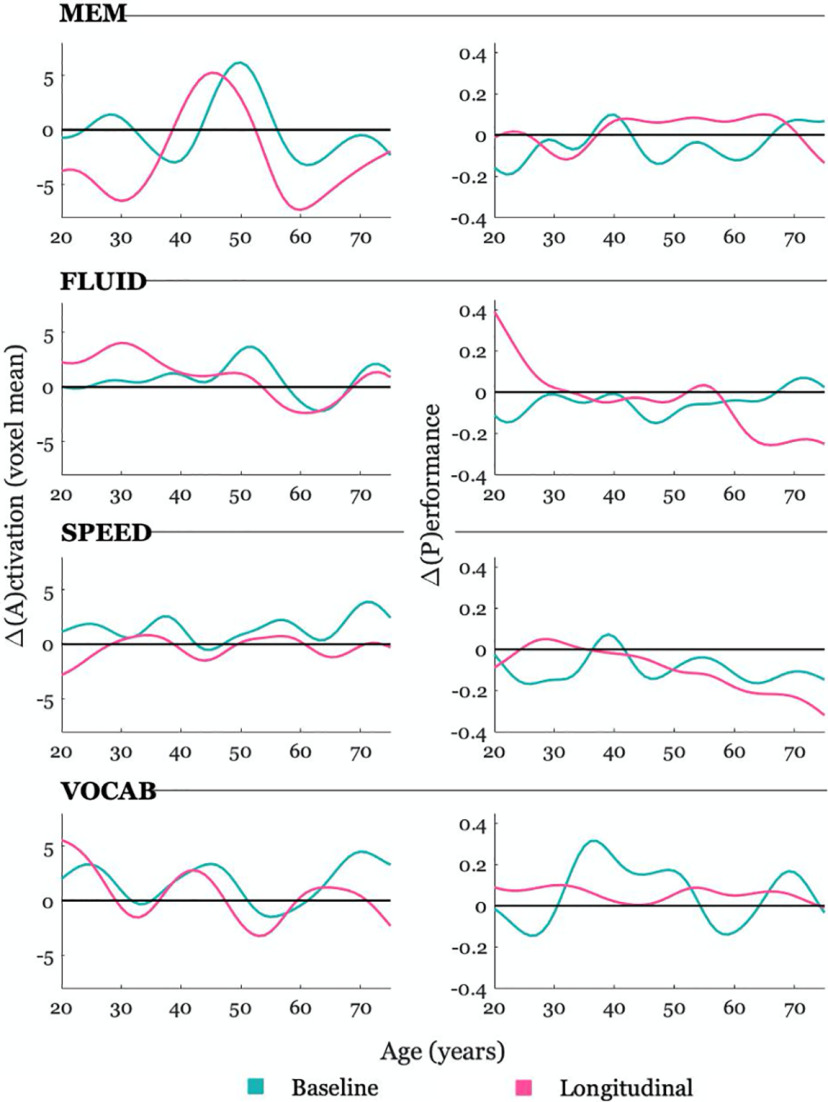
Five year change curves of baseline approximations and longitudinal measurements. Left panel, Neural change curves. The values reflect the age-weighted differences between the activation map at time (*t*) subtracted from the map at time (*t* + 5) averaged across all voxels (*y*-axis) plotted separately for each domain. Right panel, Behavioral performance change curves. The values reflect the age-weighted differences in behavioral performance at time (*t*) subtracted from performance at time (*t* + 5; *y*-axis) plotted separately for each domain. This 5 year window of difference, expressed as a single value, is plotted for the age at baseline (*x*-axis). Baseline approximations (green) and real longitudinal change measurements (pink) are plotted together to visually appreciate similarities versus discrepancies.

We first present the change curves computed by taking the average across all voxels from the change maps, for both the baseline approximation and longitudinal measurements (Fig. 2, top). We calculated the similarity between baseline and longitudinal change curves per domain using MAE, where lower values indicate greater similarity. According to MAE, the FLUID domain displayed the highest similarity between baseline and longitudinal measurements (MAE = 1.52), whereas the MEM domain displayed the highest difference (MAE = 3.82), followed by SPEED (MAE = 1.82) and VOCAB (MAE = 1.72). However, as one can appreciate from the figure, there do not appear to be radical differences between baseline and longitudinal measurements, either in shape or magnitude, with peaks occurring at similar points for each across all domains.

### Behavioral performance change curves

We next looked at the performance change curves for both baseline approximations and longitudinal measurements (Fig. 2, bottom). We again calculated MAE for the change curve comparisons per domain. According to MAE, the SPEED domain displayed the highest similarity between baseline approximations and longitudinal measurements (MAE = 0.09), whereas the FLUID domain displayed the highest difference (MAE = 0.14), followed by the VOCAB domain (MAE = 0.13) and finally the MEM domain (MAE = 0.11). Interestingly, whereas the baseline approximations of change for the VOCAB domain indicated troughs of performance decreases, notably in the change from ∼58 to 63 years of age (i.e., represented as a baseline age of 58 years on the graph), the longitudinal measurements always showed increases in performance across the life span. Overall, whereas baseline approximations tended to display consistent declines in performance over time, longitudinal measurements displayed a more variable pattern of both increases and decreases, with only SPEED showing a rather constant increase in the slope of decline.

### Baseline approximations compared with longitudinal neural change: change curves per ROI divided by age bracket

**Figure 3.**
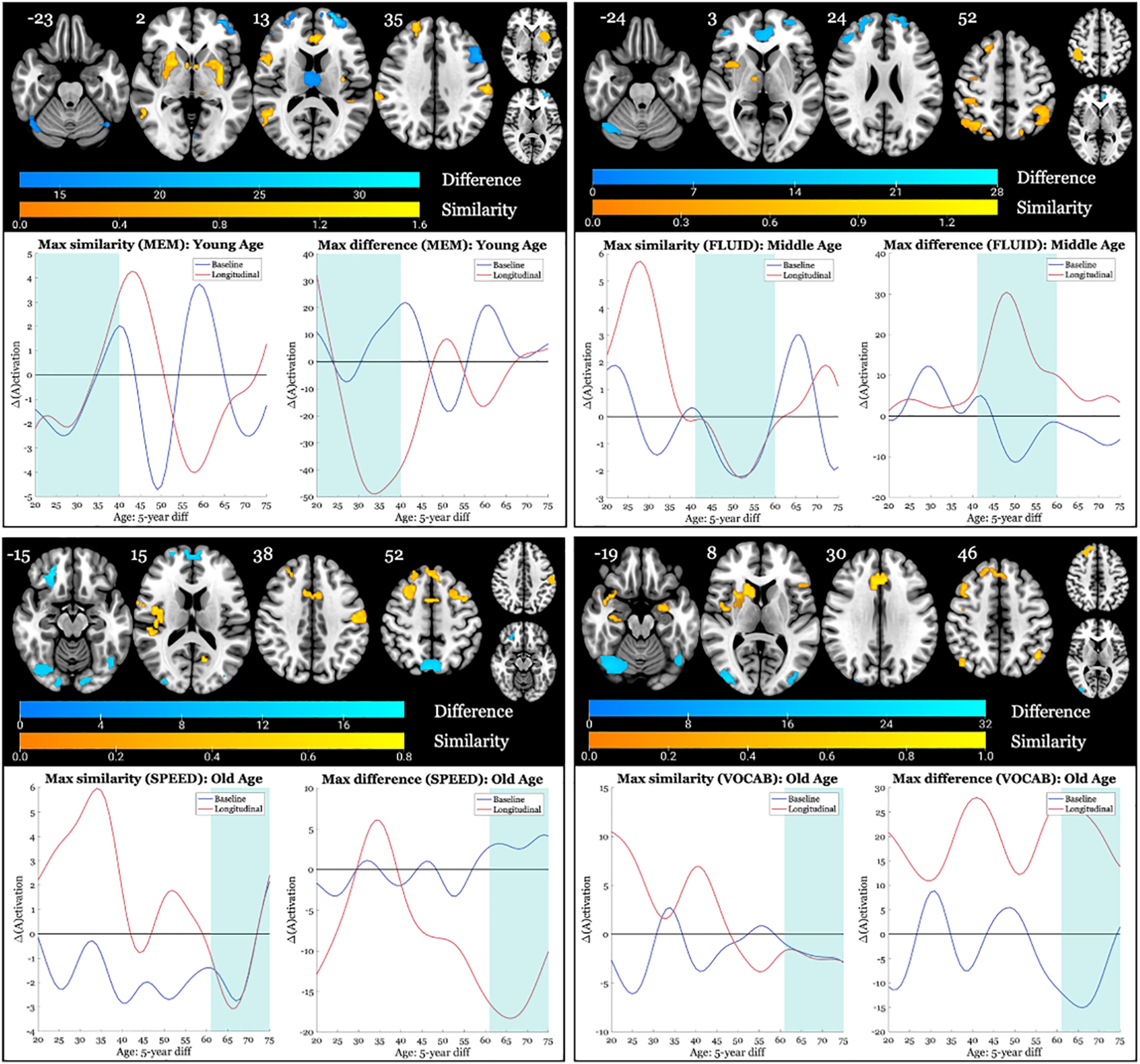
Axial brain slices expressing areas of greatest similarity and difference between baseline approximations and longitudinal measurements of change for each domain. We selected an age bracket to represent per domain. For each domain map, we display the regions for a given age bracket (indicated in the graph title) displaying both the greatest difference between curves (MAE >97.5 percentile; depicted in blue) and the greatest similarity (MAE <2.5 percentile; depicted in yellow). The number next to each brain slice indicates the *z*-coordinate. To the top right, the two smaller brain slices represent the two ROIs displaying the greatest similarity (yellow) and difference (blue) between curves, which are represented in the graphs below. Graphs depict the change in activation (*y*-axis) for each 5 year window (plotted on the *x*-axis at baseline age). The shaded blue region denotes the age bracket segment on which MAE was calculated. Note: slices are mirror flopped where the right hemisphere is expressed on the left side.

Next, we compared the baseline approximation to the longitudinal change curves for each ROI, centered on each voxel in our gray matter mask, across the entire brain, for each domain. These comparisons were made by dividing the curves into segmented age brackets that approximately represented tertiles in the age distribution at baseline and calculating MAE on these segments. We were interested in which brain areas displayed maximum similarities and differences between the two measurements, defined as <2.5 or >97.5 percentiles, respectively. An example of the map of these regions, one for each domain, can be found in Figure 3. We also list the top three ROIs expressing the greatest difference (Table 2) and the top three ROIs expressing the greatest similarity (Table 3) for each age bracket in each domain. As can be observed from the figure, the MAE provided a good approximation of similarity and difference for each of the domains presented. Among the differences, those greatest across all domains were observed in the right hemisphere for the old age bracket. Overall, it appeared that for the young and middle age brackets, the greatest differences for all domains were expressed in frontal regions, often left lateralized, including the superior and middle frontal gyri. The one exception was for the VOCAB domain, where the middle age bracket displayed highest differences in the calcarine fissure, middle occipital lobe, and the cerebellum crus 1. Conversely, the greatest differences between baseline and longitudinal measurements for the old age bracket were observed in posterior regions, such as the right inferior/middle occipital cortex, lingual gyrus, and cerebellum crus 6, and the bilateral cerebellum crus 1. Only for the SPEED domain, the orbital middle frontal gyrus was among the regions that expressed maximum difference in the old age bracket. There appeared to be less uniformity across domains among the regions expressing similarities between both measurements. However, interestingly, whereas posterior regions such as the inferior/middle occipital cortex displayed the greatest differences between measurements for the VOCAB domain in the old age bracket, anterior regions such as the bilateral superior frontal gyrus consistently showed the greatest similarity. Additionally, whereas regions expressing both maximum similarity and difference in the old age bracket were typically right lateralized, only the SPEED domain displayed left-lateralized similarity between measurements, including the inferior parietal lobule [Fig. 3, bottom left (this similarity was a common decrease in activation)]. Furthermore, more parietal regions such as the supramarginal, postcentral, and inferior parietal gyri and precuneus displayed similarity, along with limbic structures such as the caudate, putamen, and hippocampus.

**Table 2:** AAL Regions displaying the greatest difference between baseline approximations and longitudinal measurements

		Coordinates		
Domain	Age group	*x*	*y*	*z*	AAL region	Hem	MAE	N. ROIs
MEM	Young	−24	57	3	Superior frontal gyrus (DL)	L	35.65	57
		−27	54	3	Middle frontal gyrus	L	31.06	132
		−30	54	−3	Superior frontal gyrus (ORB)	L	30.96	2
	Middle	−42	−63	−24	Cerebellum crus 1	L	29.46	16
		−21	63	12	Superior frontal gyrus (DL)	L	28.64	48
		−42	−57	−24	Cerebellar crus 6	L	28.41	56
	Old	30	−84	−18	Lingual gyrus	R	39.74	94
		36	−84	−15	Inferior occipital lobe	R	37.86	56
		27	−84	−18	Cerebellar crus 1	R	37.58	47
FLUID	Young	−21	63	9	Superior frontal gyrus (DL)	L	28.22	61
		−9	63	12	Superior frontal gyrus (Med)	L	21.89	157
		−27	57	12	Middle frontal gyrus	L	19.84	64
	Middle	−9	45	−9	Superior frontal gyrus (MedOrb)	L	22.64	10
		−6	33	−6	Cingulate gyrus (Ant)	L	21.20	73
		6	39	−9	Superior frontal gyrus (MedOrb)	R	20.71	9
	Old	39	−69	−24	Cerebellar crus 1	R	20.15	63
		−42	−63	−24	Cerebellar crus 1	L	18.64	16
		36	−72	−21	Cerebellar crus 6	R	17.19	111
SPEED	Young	−45	45	−9	Inferior frontal gyrus (ORB)	L	21.61	30
		−42	45	−6	Middle frontal gyrus (Orb)	L	20.05	18
		36	54	6	Middle frontal gyrus	R	18.70	105
	Middle	−30	54	−3	Superior frontal gyrus (Orb)	L	18.49	2
		−33	54	−3	Middle frontal gyrus (Orb)	L	17.99	18
		18	−93	−12	Lingual gyrus	R	16.02	49
	Old	24	36	−21	Middle frontal gyrus (Orb)	R	19.17	8
		36	−90	0	Inferior occipital lobe	R	18.75	52
		36	−90	3	Middle occipital lobe	R	18.66	42
VOCAB	Young	−39	51	−6	Middle frontal gyrus (Orb)	L	33.88	18
		−30	54	−3	Superior frontal gyrus (Orb)	L	33.36	2
		−33	54	0	Superior frontal gyrus (DL)	L	32.88	40
	Middle	30	−90	9	Middle occipital lobe	R	23.33	88
		15	−96	3	Calcarine fissure + surrounding cortex (V1)	R	21.23	23
		39	−69	−24	Cerebellar crus 1	R	20.26	51
	Old	36	−90	3	Middle occipital lobe	R	32.27	103
		36	−90	0	Inferior occipital lobe	R	30.82	92
		39	−69	−24	Inferior occipital lobe	R	24.29	27

The three regions per age bracket and domain displaying the greatest difference, via MAE metric at the >97.5 percentile, are presented. Coordinates refer to the center voxel of the ROI. As MAE is a negative-oriented error metric, higher values indicate higher differences. The “N.ROIs” column represents the number of ROIs in the >97.5 percentile subset (601 ROIs/comparison) for which the left voxel is located in the AAL (automated anatomical labeling) region listed. For instance, in the case of the first row entry, the left dorsolateral superior frontal gyrus displayed the greatest difference at *x*, *y*, and *z* locations (−24, 57, and 3), but this region was among the top 601 ROIs displaying the greatest differences for 57 of the 601 ROIs. Hem, Hemisphere; L, left; R, right; DL, dorsolateral; Orb, orbital; Med, medial.

**Table 3:** ROIs Denoted by AAL area displaying the greatest similarity between baseline approximations and longitudinal measurements

		Coordinates		
Domain	Age group	*x*	*y*	*z*	AAL region	Hem	MAE	N. ROIs
MEM	Young	−27	3	0	Putamen	L	0.42	109
		15	−6	66	Superior frontal gyrus (DL)	R	0.47	27
		51	−39	54	Inferior parietal gyrus	R	0.49	43
	Middle	15	−84	39	Cuneus	R	1.28	26
		42	−51	57	Superior parietal gyrus	R	1.58	17
		−6	−69	48	Precuneus	L	1.65	72
	Old	63	−33	24	Superior temporal gyrus	R	0.22	92
		60	−33	27	Supramarginal gyrus	R	0.28	80
		42	21	48	Middle frontal gyrus	R	0.39	41
FLUID	Young	−30	12	−9	Undefined	L	0.37	111
		48	−75	30	Middle occipital lobe	R	0.38	31
		−12	6	63	Supplementary motor area	L	0.38	11
	Middle	36	−36	54	Postcentral Gyrus	R	0.21	14
		39	−66	42	Angular Gyrus	R	0.23	105
		30	9	9	Putamen	R	0.27	14
	Old	33	−12	−27	Parahippocampal	R	0.33	28
		21	24	60	Superior frontal gyrus (DL)	R	0.39	51
		36	−15	−21	Hippocampus	R	0.42	18
SPEED	Young	6	−60	36	Precuneus	R	0.29	96
		39	21	−6	Insula	R	0.36	28
		−18	−33	6	Undefined	L	0.37	24
	Middle	12	15	60	Supplementary motor area	R	0.24	35
		66	−27	15	Superior temporal gyrus	R	0.28	56
		30	9	51	Middle frontal gyrus	R	0.28	69
	Old	−57	−18	45	Inferior parietal gyrus	L	0.23	5
		−3	12	36	Cingulate gyrus (Mid)	L	0.26	26
		−24	12	51	Middle frontal gyrus	L	0.32	44
VOCAB	Young	−6	−3	−9	Undefined	L	0.34	138
		−15	3	15	Caudate	L	0.52	59
		15	0	18	Caudate	R	0.95	34
	Middle	−6	18	30	Cingulate gyrus (Ant)	L	0.34	36
		3	18	27	Cingulate gyrus (Ant)	R	0.37	22
		−45	6	30	Inferior frontal gyrus (opercular)	L	0.44	60
	Old	18	36	48	Superior frontal gyrus (DL)	R	0.22	49
		−6	30	48	Superior frontal gyrus (Med)	L	0.27	32
		9	33	51	Superior frontal gyrus (Med)	R	0.27	42

The three regions per age bracket and domain displaying the greatest similarity, via MAE metric at the <2.5 percentile, are presented. Coordinates refer to the left voxel of the ROI. As MAE is a negative-oriented error metric, lower values indicate higher similarity. The “N.ROIs” column represents the number of ROIs in the <2.5 percentile subset (601 ROIs per comparison) for which the center voxel is located in the AAL (automated anatomical labeling) region listed. For instance, in the case of the first row entry, the left putamen displayed the greatest similarity at *x*, *y*, and *z* locations (−27, 3, and 0), but this region was among the top 601 ROIs displaying the greatest differences for 109 of the 601 ROIs. Hem, Hemisphere; L, Left; R, right; DL, dorsolateral; Med, medial; Ant, anterior; Mid, middle.

### Longitudinal changes across the life span

#### Integrated change by age bracket

**Figure 4.**
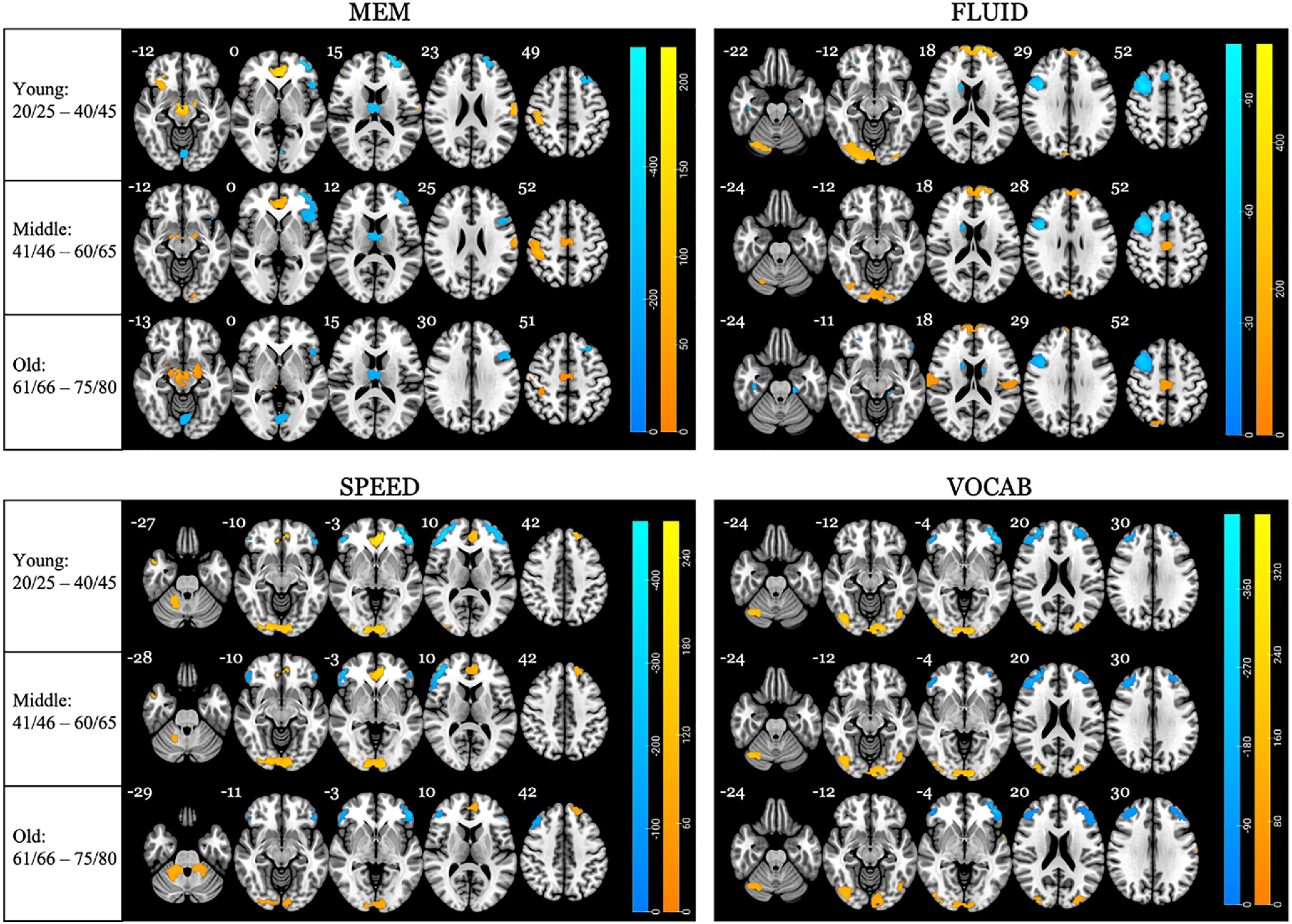
Areas of maximum change in longitudinal measurements. The brain regions displaying the greatest integrated positive change at >97.5 percentile (yellow) and the greatest integrated negative change (blue) are presented for each age bracket in each domain. The color bars to the right of each image reflect the scale of change for each domain. The extreme ends of the scale were chosen based on the maximum and minimum change values observed across all age brackets; these values were always greatest for the young age bracket. The number next to each brain slice indicates the *z*-coordinate. Note: slices are mirror flopped where the right hemisphere is expressed on the left side.

We wanted to establish which areas exhibited maximum change, in terms of both increases and decreases in activation, focusing now only on the longitudinal measurements. This was achieved by calculating the integral of both negative and positive change values in each age bracket of each ROI and domain. We then selected the extreme ends of the distribution, or values >97.5 percentile and <2.5 percentile (Fig. 4; for all four domains, both the maximum negative and positive values were highest for the young age bracket, which decided the extreme ends of the color bars). The top three areas expressing maximum positive change and maximum negative change, for each age bracket and domain, are listed in Tables 4 and 5, respectively. Overall, there were more cumulative positive changes than negative changes for the FLUID domain, as can be observed from the color bar of the graphs; this stood in contrast to MEM, for which change was overall more negative. For the MEM domain, the anterior cingulate expressed maximum increases in activation in both the young and middle age brackets but not in the old age bracket. In a similar vein, the left superior frontal gyrus, which expressed maximum decreases in activation in both the young and middle age brackets, was not present for the old age bracket. For the FLUID domain, the right cerebellum was among the regions expressing maximum activation increases in the young age bracket, but were not among the regions of highest positive change in the middle and old age brackets; a similar finding was observed for the left medial superior frontal gyrus. Conversely, the bilateral postcentral and Rolandic operculum were among the regions of highest positive change only for the old age bracket. For the SPEED domain, similar to the MEM domain, the anterior cingulate cortex (ACC) expressed maximum increases in activation in the young and middle age brackets but to a reduced extent in the old age bracket. Instead, the bilateral cerebellum crura 3–6 displayed maximum increases in activation in the old age bracket, which was not among the top regions expressing change in the young and middle age brackets. Furthermore, maximum decreases in activation in the medial/superior frontal gyrus, which were present in the young and middle age brackets, were present to a lesser degree in the old age bracket, with the latter expressing maximum decreases in more left-lateralized inferior frontal operculum. For the VOCAB domain, the most salient finding was the stability in the expression of maximum change across all age brackets, with maximum positive changes consistently occurring in posterior regions such as the inferior/middle occipital lobe, and maximum negative changes occurring in frontal regions such as the inferior/middle frontal gyrus.

**Table 4:** ROIs Denoted by AAL area expressing the greatest integrated positive change for longitudinal measurements

		Coordinates		
Domain	Age group	*x*	*y*	*z*	AAL region	Hem	Integral	N. ROIs
MEM	Young	−3	30	−6	Cingulate gyrus (Ant)	L	220.34	71
		3	33	−6	Cingulate gyrus (Ant)	R	213.26	35
		9	36	−9	Superior frontal gyrus (MedOrb)	R	212.14	9
	Middle	−3	42	−6	Superior frontal gyrus (MedOrb)	L	142.20	10
		−9	48	−3	Cingulate gyrus (Ant)	L	141.01	66
		3	33	−6	Cingulate gyrus (Ant)	R	136.41	32
	Old	15	−15	−24	Undefined	R	132.92	302
		15	−9	−15	Hippocampus	R	99.99	9
		−15	−6	−12	Hippocampus	L	93.77	41
FLUID	Young	−21	63	9	Superior frontal gyrus (DL)	L	534.51	49
		−9	63	12	Superior frontal gyrus (Med)	L	388.11	85
		−27	57	12	Middle frontal gyrus (MedOrb)	L	369.12	57
	Middle	−21	63	9	Superior frontal gyrus (DL)	L	340.66	41
		−9	63	12	Superior frontal gyrus (Med)	L	282.29	91
		15	−93	−12	Lingual gyrus	R	241.39	30
	Old	24	−93	−12	Lingual gyrus	R	254.76	18
		27	−90	−9	Inferior occipital lobe	R	221.99	23
		15	63	15	Superior frontal gyrus (Med)	R	188.51	46
SPEED	Young	−6	45	−9	Superior frontal gyrus (MedOrb)	L	265.70	10
		−6	39	−6	Cingulate gyrus (Ant)	L	236.82	105
		6	39	−9	Superior frontal gyrus (MedOrb)	R	223.88	8
	Middle	−6	45	−6	Superior frontal gyrus (MedOrb)	L	204.89	10
		18	−96	−3	Calcarine fissure + surrounding cortex (V1)	R	201.23	31
		−9	48	−3	Cingulate gyrus (Ant)	L	194.51	97
	Old	24	−42	−33	Undefined	R	117.47	145
		18	−96	−3	Calcarine fissure + surrounding cortex (V1)	R	111.69	14
		−6	51	6	Superior frontal gyrus (Med)	L	105.11	39
VOCAB	Young	30	−90	9	Middle occipital lobe	R	375.25	88
		15	−96	3	Calcarine fissure + surrounding cortex (V1)	L	355.38	31
		33	−90	0	Inferior occipital lobe	R	336.67	85
	Middle	30	−90	9	Middle occipital lobe	R	349.44	88
		15	−96	3	Calcarine fissure + surrounding cortex (V1)	R	333.87	31
		33	−90	0	Inferior occipital lobe	R	310.89	81
	Old	15	−96	3	Calcarine fissure + surrounding cortex (V1)	R	252.60	30
		30	−90	9	Middle occipital lobe	R	242.30	71
		33	−90	0	Inferior occipital lobe	R	205.04	79

The three regions per age bracket and domain displaying the greatest integrated positive change at the >97.5 percentile, are presented. Integrated change was calculated via trapezoidal summation in the segmented age bracket. Coordinates refer to the left voxel of the ROI. Higher values signify greater positive change. The “N.ROIs” column represents the number of ROIs in the >97.5 percentile subset (601 ROIs/comparison) for which the center voxel is located in the AAL (automated anatomical labeling) region listed. For instance, in the case of the first row entry, the left anterior cingulate gyrus displayed the greatest positive change at *x*, *y*, and *z* locations (−3, 30, and −6), but this region was among the top 601 ROIs displaying the greatest positive change for 71 of the 601 ROIs. Hem, Hemisphere; L, left; R, right; DL, dorsolateral; Med, medial; Ant, anterior; Med, medial.

**Table 5:** ROIs Denoted by AAL area expressing the greatest integrated negative change for longitudinal measurements

		Coordinates		
Domain	Age group	*x*	*y*	*z*	AAL region	Hem	Integral	N. ROIs
MEM	Young	−24	57	3	Superior frontal gyrus (DL)	L	−580.56	69
		−27	54	3	Middle frontal gyrus	L	−523.74	179
		−30	54	−3	Superior frontal gyrus (Orb)	L	−516.44	2
	Middle	−30	54	−3	Superior frontal gyrus (Orb)	L	−445.79	2
		−33	54	−3	Middle frontal gyrus (Orb)	L	−405.77	18
		−30	54	3	Middle frontal gyrus	L	−379.80	115
	Old	−51	15	36	Inferior frontal gyrus (opercular)	L	−294.61	30
		−48	18	39	Middle frontal gyrus	L	−288.31	78
		−51	12	39	Precentral gyrus	L	−269.83	23
FLUID	Young	36	9	60	Middle frontal gyrus	R	−105.61	269
		51	12	42	Precentral gyrus	R	−82.72	77
		51	15	39	Inferior frontal gyrus (opercular)	R	−78.94	110
	Middle	36	9	60	Middle frontal gyrus	R	−100.77	266
		51	12	42	Precentral gyrus	R	−78.12	83
		51	15	39	Inferior frontal gyrus (opercular)	R	−74.91	104
	Old	45	15	48	Middle frontal gyrus	R	−72.82	277
		39	−21	−27	Fusiform	R	−64.45	18
		51	18	39	Inferior frontal gyrus (opercular)	R	−63.05	107
SPEED	Young	−42	45	−9	Inferior frontal gyrus (Orb)	L	−471.46	38
		−42	45	−6	Middle frontal gyrus (Orb)	L	−452.86	18
		36	51	6	Middle frontal gyrus	R	−400.16	176
	Middle	45	45	6	Middle frontal gyrus	R	−317.70	172
		42	45	0	Inferior frontal gyrus (triangular)	R	−292.00	236
		42	45	−3	Inferior frontal gyrus (Orb)	R	−278.74	53
	Old	−45	45	−9	Inferior frontal gyrus (Orb)	L	−297.48	53
		−48	39	0	Inferior frontal gyrus (triangular)	L	−274.64	47
		45	45	6	Middle frontal gyrus	R	−222.84	187
VOCAB	Young	−42	45	−9	Inferior frontal gyrus (Orb)	L	−445.70	28
		−39	48	−6	Middle frontal gyrus (Orb)	L	−424.14	18
		−42	51	3	Middle frontal gyrus	L	−346.89	148
	Middle	36	51	6	Middle frontal gyrus	R	−324.85	185
		−42	51	6	Middle frontal gyrus	L	−251.18	178
		−42	48	6	Inferior frontal gyrus (triangular)	L	−243.30	44
	Old	−42	45	−9	Inferior frontal gyrus (Orb)	L	−320.47	40
		−42	45	−6	Middle frontal gyrus (Orb)	L	−286.34	18
		−48	42	0	Inferior frontal gyrus (triangular)	L	−224.47	187

The three regions per age bracket and domain displaying the greatest integrated negative change at the <2.5 percentile, are presented. Integrated change was calculated via trapezoidal summation in the segmented age bracket. Coordinates refer to the left voxel of the ROI. Lower values signify greater negative change. The “N.ROIs” column represents the number of ROIs in the <2.5 percentile subset (601 ROIs per comparison) for which the center voxel is located in the AAL (automated anatomical labeling) region listed. For instance, in the case of the first row entry, the left dorsolateral superior frontal gyrus displayed the greatest negative change at *x*, *y*, and *z* locations (−27, 57, and 3), but this region was among the top 601 ROIs displaying the greatest negative change for 69 of the 601 ROIs. Hem, Hemisphere; L, left; R, right; DL, dorsolateral; Med, medial; Ant, anterior; Med, medial.

#### Peak longitudinal change across the life span

**Figure 5.**
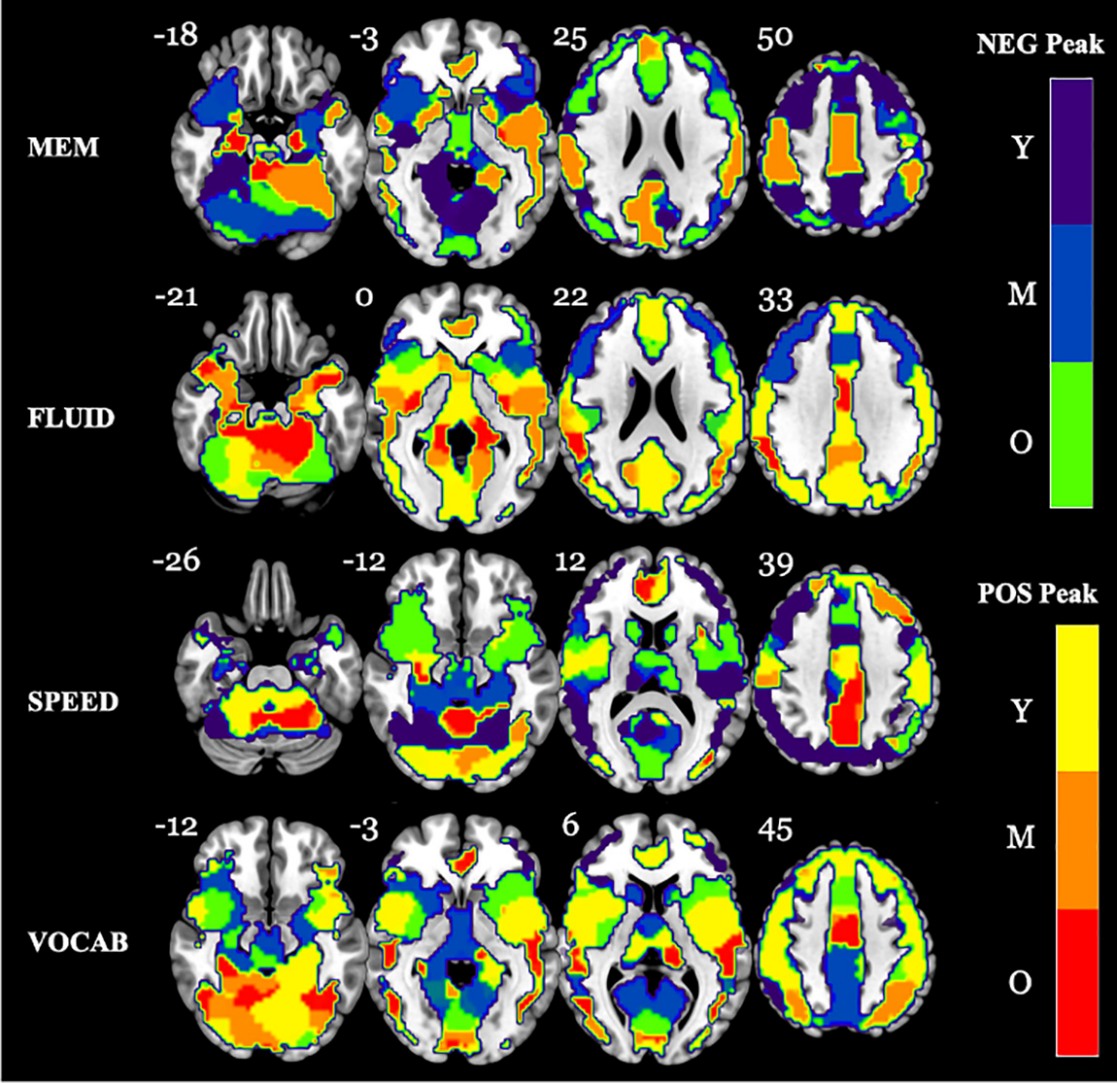
Domain maps depicting the age bracket in which peak negative or positive changes occurred across the entire life span change curve. For each ROI in each domain, we located the overall peak change value, regardless of sign, across the entire change curve (essentially the maximum absolute value). The center voxel of each ROI was then color coded depending on the age bracket in which the peak was located and whether it was a positive or negative peak (see color bar to the right of the figure). The number next to each brain slice indicates the *z*-coordinate. Note: slices are mirror flopped where the right hemisphere is expressed on the left side. Y, Young; M, middle age; O, old.

We wished to establish in which age bracket a peak change occurred when considering the entire change curve. Therefore, for each ROI in a given domain, we indexed the maximum value of the absolute value of the change curve and assigned it a color label based on the age bracket in which it occurred and the original sign of the peak (negative or positive; Fig. 5). For the MEM domain, it was clear that the bilateral (para)hippocampus and cerebellar vermis lobules 1–3 displayed the highest increases in activation for the older age bracket, whereas areas such as bilateral thalamus, anterior cingulate, cerebellar vermis lobules 4–6, and middle occipital lobe displayed peak decreases in activation. However, it appeared that, overall, the maximum changes were occurring for the middle age bracket, in terms of both peak increases and decreases in activation, with a slight left hemispheric bias in the medial/superior temporal lobe and cerebellum crura 4 and 5 toward peak increases in activation; additionally, the precuneus, cuneus, and supplementary motor area displayed peak increases, whereas the bilateral cerebellar crus 6, insula, and inferior frontal gyrus (pars orbitalis) and right superior temporal pole displayed peak decreases. For the FLUID domain, as could be expected from the integrated change analysis, the maximum changes were mainly positive peaks, with broad areas of the bilateral temporal lobe and midline extending from the cuneus to the anterior cingulate expressing positive peaks in middle age and young age brackets, respectively. Positive peaks were seen in the left fusiform, bilateral cerebellum, bilateral (para)hippocampus, right supramarginal gyrus, midcingulate, and bilateral putamen. Negative peaks in middle age were mainly observed bilaterally along the rostrocaudal axis of the prefrontal cortex. For the SPEED domain, greater peak decreases in activation were observed for young age bracket. Interestingly, some of these peaks were located in the right inferior parietal lobule and angular gyrus, areas that, along with their left counterparts, have been implicated in attention and action guidance (Singh-Curry and Husain, 2009). Other areas of peak activation decreases in the young were the bilateral fusiform and lingual gyri, bilateral inferior/middle temporal lobe, and bilateral inferior frontal triangularis as well as the middle frontal gyrus. Peak decreases were observed for old age in the vast regions of the bilateral putamen, superior temporal pole, supplementary motor area, insula, Rolandic operculum, and orbital inferior frontal gyrus, whereas peak increases in activation were observed in the precuneus, midcingulate, and primarily left cerebellar crura 4 and 5, and right anterior cingulate. For the VOCAB domain, there were large peak increases in activation for the young age bracket along the midline from the medial superior frontal to the posterior cingulate cortex and bilateral along the precentral and postcentral gyri. The old age bracket also displayed peak activation increases in a portion of the midcingulate in addition to the bilateral inferior/medial temporal cortex and fusiform, the left superior temporal cortex, the right cerebellar crura 4–6, and primarily the left anterior cingulate. Peak decreases in activation were mainly found for the middle age bracket and extended through large portions of the bilateral cuneus, precuneus, calcarine, lingual gyrus, and posterior cingulate. Decreases in the precuneus were also observed for the young age bracket. While several regions maintained the same sign of peak change, only differing in age bracket, a few stood out for flipping signs between domains. For instance, whereas the bilateral vermis lobules 4 and 5, the posterior portion of the right anterior cingulate, and the left middle frontal gyrus all displayed peak decreases in activation for MEM in the old, they displayed peak increases in activation in the old for SPEED. In addition, posterior regions belonging to the bilateral middle occipital lobe, cuneus, and angular gyri that displayed peak increases in activation in the young age bracket for the FLUID domain instead displayed peak decreases in activation for the SPEED domain for the same age bracket.

#### Stability of longitudinal change across domains.

**Figure 6.**
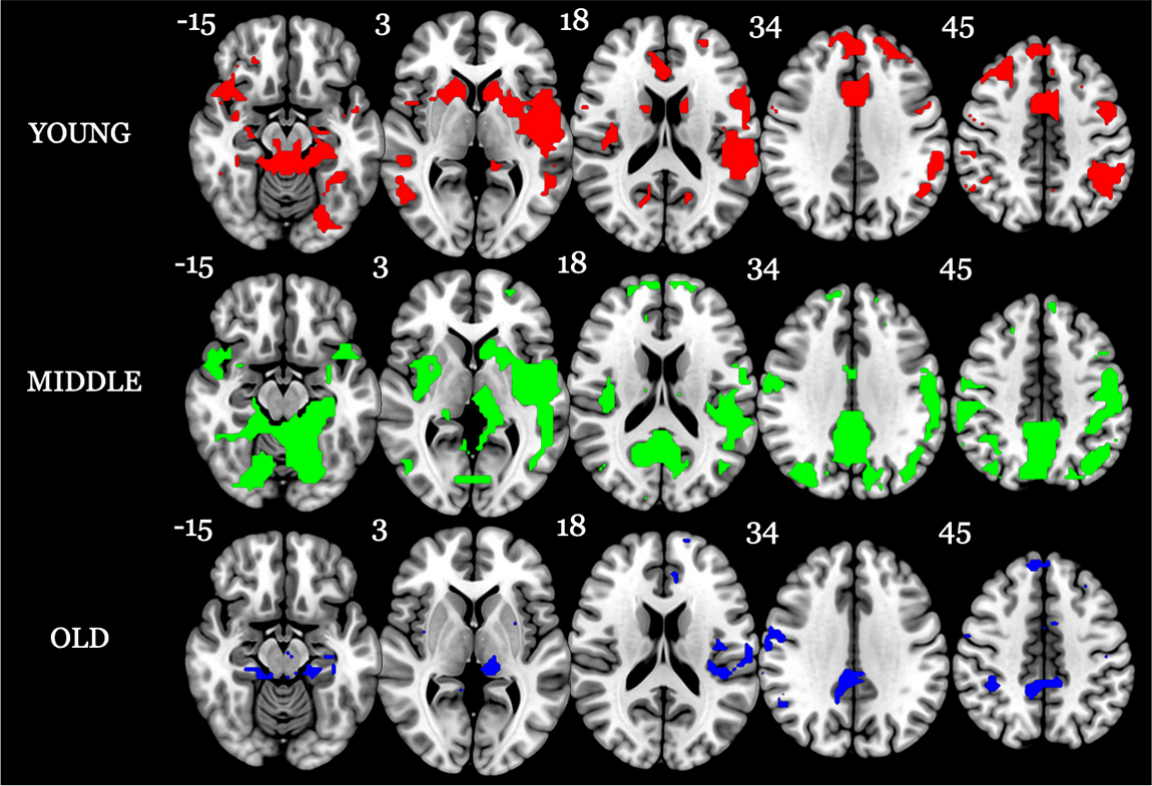
Maps of each age bracket depicting regions of sign change present in all four domains. For each domain, we indexed the ROIs in which the change curve contained at least one zero crossing, denoting a sign change (i.e., positive–negative or negative–positive). We then selected those ROIs that displayed overlap in sign change across all four domains. These voxels are mapped separately for the young (red), middle (green), and old (blue) age brackets. The number next to each brain slice indicates the *z*-coordinate. Note: slices are mirror flopped where the right hemisphere is expressed on the left side.

As a final analysis, we wished to measure the stability of longitudinal change across domains in each age bracket, defined as voxels expressing at least one sign change (positive–negative or negative–positive) in each of the four domains [Fig. 6, display of these regions (colored regions display fluctuations, whereas white regions display constant sign change in at least one domain)]. As can be observed from the figure, all age brackets contained regions expressing change of a constant sign, in either the negative or positive direction, at least once in all four cognitive domains. In terms of regions of sign fluctuations, both the young and middle age bracket displayed change fluctuations in slightly left-lateralized regions such as the caudate, putamen, Rolandic operculum, insula, and superior temporal pole. The young age bracket displayed further fluctuations in anterior regions including the anterior and midcingulate, whereas the middle age bracket displayed sign fluctuations in regions including the precuneus and posterior cingulate. However, perhaps the most striking finding occurred in the old age bracket, where only a few regions displayed sign fluctuations present in all four domains; that is, the greatest stability in direction of change was witnessed in the old age bracket. Among those regions with expression change were the right cerebellar crura 4 and 5, right postcentral gyrus, bilateral medial cingulate and precuneus, and left thalamus.

## Discussion

The aim of the present study was to quantify and compare cross-sectional approximations to longitudinal measurements of change across the life span and to further probe characteristics of this change in time (i.e., age) and space (i.e., ROI regions) specifically in longitudinal measurements. To this end, we tested participants in-scanner on a battery of cognitive tasks at two time points and used both behavioral performance and neural voxel activations to quantify continuous change across the life span. In a preliminary comparison of voxel-averaged neural change curves between cross-sectional and longitudinal measurements, we showed that change curves did not greatly differ, in either shape or magnitude, across domains. However, this was performed simply to gain a first impression of our data, as coarse whole-brain voxel-averaged change is not typically considered, possessing dubious ecological validity and being potentially uninformative in washing out nuanced effects. When we computed age-weighted ROI activation maps, region-specific change curves, instead, showed varying similarity between the two measurements. A further division of each curve into age brackets and comparison between measurements revealed areas displaying high dissimilarity. We further identified regions of maximum positive and negative change for each domain and age bracket in longitudinal measurements only, and were interested in the topography of when peak changes occurred across the life span.

The majority of what we know concerning age-related neural and cognitive changes comes from cross-sectional studies, despite limitations of potential cohort effects confounding true age-specific changes. Cross-sectional comparisons of different ages have generally shown negative associations between age and performance on several cognitive abilities (Salthouse, 2009). However, longitudinal evidence has shown a different pattern of change, with sustained or even increased performance into later life (Ronnlund et al., 2005; Schaie and Willis, 2010; Salthouse, 2014b). Our current results comparing cross-sectional to longitudinal change reflect these discrepancies; whereas cross-sectional approximations of change mainly displayed performance declines across the life span, except for the vocabulary domain, longitudinal measurements displayed periods of stable increases in performance across the life span. The notable exception was the processing speed domain, for which declines were observed beginning at ∼35 years of age, with the steepness of decline increasing with age. This latter finding has also been observed in a recent longitudinal study on midlife cognitive changes (Hughes et al., 2018).

In terms of age-related neural changes, one of the most reported cross-sectional findings is the increase in activation in frontal brain regions, which has often been interpreted as a compensatory response to counteract neurocognitive decline (Davis et al., 2008; Drag and Bieliauskas, 2010). Interestingly, when comparing age-bracketed segments of the change curves between cross-sectional and longitudinal measurements, we observed that the maximum differences for all domains were expressed in predominantly left-lateralized frontal regions among young and middle age brackets. In the old age bracket, maximum differences were observed in more posterior regions, including the right occipital cortex, lingual gyrus, and bilateral cerebellum. When looking at maximum integrated change by age bracket in the longitudinal measurements, we further see that negative changes, or activation declines, were predominantly present in inferior, middle, and superior frontal regions across all age brackets. Conversely, maximum integrated positive change showed a more variable pattern across age brackets and domains, with the vocabulary domain showing the highest stability across all age brackets; importantly, maximum negative change occurred in frontal regions such as the inferior/middle frontal gyrus, whereas maximum positive change occurred in posterior regions such as the inferior/middle occipital lobe. This latter finding is particularly notable as vocabulary is a cognitive ability that shows improvement with age (Salthouse and Davis, 2006; Hartshorne and Germine, 2015), additionally observed in our own data. While we cannot infer that improved behavioral performance is linked to neural changes in the regions listed above, recruitment of frontal resources to maintain or increase behavioral outcomes may not strictly apply to all cognitive domains and should be confirmed in longitudinal data. However, our findings more generally suggest that age-related increases in frontal regions reported in cross-sectional analyses may not adequately reflect true longitudinal neural changes. Even in terms of absolute change values between age brackets, the old age bracket expressed the lowest positive change values across all four domains, eliminating the possibility that frontal regions, while still overall higher for the old age bracket, were simply excluded by our threshold. Some work has highlighted the importance of characterizing the magnitude of BOLD response in terms of relative activation change when comparing younger to older adults (Spreng et al., 2010), showing that while some regions may be lower for older adults, the summation of BOLD response across all regions and trials does not differ between groups (Buckner et al., 2000). Our findings suggest that frontal regions do not display over-recruitment, either in relative change between regions within the old age bracket or in absolute change between age brackets. While ample cross-sectional evidence exists supporting increased frontal recruitment with age across different cognitive domains (Cabeza, 2002; Milham et al., 2002; Turner and Spreng, 2012; Hakun et al., 2015a), some longitudinal evidence suggests the under-recruitment of frontal regions, specifically on a semantic judgment task (Nyberg et al., 2010). Other longitudinal PET findings have reported both reductions and increases in cerebral blood flow across prefrontal cortex regions when performing verbal and figure recognition tasks (Beason-Held et al., 2008a,b). However, longitudinal findings are equivocal, with yet other evidence echoing claims of frontal over-recruitment, particularly when assessing executive tasks (Hakun et al., 2015b). Furthermore, one crucial aspect that is not covered by our analysis is how changes in performance relate to age-related increases or declines in activation. For instance, a longitudinal study by Vidal-Piñeiro et al. (2019) found that low levels of frontal activation during an episodic memory task was associated with lower memory performance in older adults over an 8 year period. More longitudinal work is needed to assess the role of frontal cortical regions in the aging process.

In addition to our findings suggesting a lack of support for age-related frontal increases as measured by maximum integrated change, the analysis of peak change across age brackets again revealed more posterior regions displaying peak increases in activation in the old age bracket. In all four domains, portions of the cerebellum, including the vermis, displayed peak positive changes in the old age bracket. A cross-sectional review by Bernard and Seidler (2014) reported task-related increases in cerebellar activation with age, particularly in motor learning and execution tasks, arguing that cerebellar morphology is comparable if not better than the prefrontal cortex at predicting performance. For the memory domain, one of the few areas displaying peak increases in activation among the old age bracket was the bilateral hippocampus. Both cross-sectional and longitudinal work has found age-related hyperactivation in the hippocampus, which has been linked to factors such as declines in memory performance and amyloid and tau accumulation (Leal et al., 2017; Huijbers et al., 2019). Peak decreases in activation were otherwise observed in the old age bracket, primarily bilaterally along the inferior–superior axis of the frontal cortex, and posteriorly in the medial occipital cortex and calcarine. However, the majority of both peak increases and decreases in activation occurred in the middle age bracket, where peak increases in activation were found in slightly left-lateralized regions of the medial/superior temporal lobe and cerebellar crura 4 and 5, and peak decreases in activation found in the bilateral cerebellar crus 6, insula, and right superior temporal pole. Limited longitudinal evidence has shown that memory performance during midlife can predict an individual’s memory-related BOLD response 15–20 years later (Pudas et al., 2014) and that the difference between an individual’s chronological age and biological age, as predicted from machine-learning models, is associated with cognitive function in early life and adulthood (Elliott et al., 2019). These studies highlight the need that greater focus be placed on this under-represented interval in the life span.

One additional region that stood out in both analyses of maximum longitudinal change and the distribution of peak change across the life span was the ACC. In both the memory and processing speed domains, the ACC expressed maximum increases in activation in the young and middle age brackets, but maximum increases were not present in the old age bracket for memory and to a reduced extent for processing speed. However, when looking at when peak positive change occurs across the life span, we see that positive peaks were in fact observed for the old age bracket in the right ACC for the speed domain and in the left ACC for the vocabulary domain. Prior cross-sectional and longitudinal work have both reported reduced metabolic uptake with age (Pardo et al., 2007, 2020), and that this reduction correlates with cognitive decline (Pardo et al., 2007). These findings encourage further work on how task-related activation changes in the ACC relate to aging.

Finally, we looked at regions that expressed fluctuation in the direction of change in each age bracket. The most striking finding was that the old age bracket displayed the least sign fluctuation in change across all four domains. This was an interesting finding, as we might have expected greater instability given that aging is typically related to increased intraindividual and interindividual variability in neural response because of a broad range of factors (Caspers et al., 2014) reduced neural selectivity for stimuli (for review, see Koen and Rugg, 2019). However, it should be highlighted that we measured stability as fluctuation in the directionality of change across all four domains. It could well be the case that certain domains might express change in a specific direction in old age, whereas others do not, a possibility precluded by the current analysis.

One potential criticism of the current study is the lack of statistical inference of the regions involved in the processing of each domain. We did not restrict comparisons between cross-sectional and longitudinal change to voxels deemed significant by univariate analysis, instead choosing to focus on activation change in a continuous manner across participants and treating all voxels as reflecting true signal. We do believe though that application of the age-weighted kernel, while by no means a rigorous statistical test, is sufficient at smoothing over nonuniform change that could have arisen because of statistical noise. We have no reason to believe that certain voxels were subject to systematic biases, given that spatial smoothing was also performed in preprocessing and that participants with a high number of motion artifact were excluded from the analysis. However, in addition to our modest sample size, we do acknowledge that the regions we report in each domain may not be “selective” to that domain with the inferential rigor of a formal statistical test. In a future application, it might be profitable to refine threshold setting across domains or measure covariance patterns of change to be able to more adequately assess unique versus overlapping change across domains.

Another future direction will be the integration of other factors associated with cognitive and neural changes across the life span. One important factor, which has formed the crux of age-related changes in the majority of longitudinal studies and reviews, has been age-related cerebral volume changes (for review, see Hedman et al., 2012). For instance, some studies have linked age-related structural brain reductions to increased functional activation (Hakun et al., 2015b; Fjell et al., 2016). Additionally, we could focus on a proper integration of brain–cognition relations, beyond simple over-recruitment of frontal activation, for better clarification of whether potential over-recruitment is linked to successful compensatory processes (Vallesi et al., 2011), as manifested by maintained or increased age-related behavioral outcomes, or by inefficiency of processing as the brain attempts to cope with negative age-related change.

## Synthesis

Reviewing Editor: Alexander Soutschek, Ludwig-Maximilians-Universität München Decisions are customarily a result of the Reviewing Editor and the peer reviewers coming together and discussing their recommendations until a consensus is reached. When revisions are invited, a fact-based synthesis statement explaining their decision and outlining what is needed to prepare a revision will be listed below. Note: If this manuscript was transferred from JNeurosci and a decision was made to accept the manuscript without peer review, a brief statement to this effect will instead be what is listed below.

The authors have successfully addressed all concerns of the reviewers. As the reviewers raised no further concerns, the manuscript is ready for publication in its current form.
